# Sample preparation of free sterols from vegetable oils by countercurrent chromatography in co-current mode

**DOI:** 10.1007/s00216-023-04766-9

**Published:** 2023-06-07

**Authors:** Felix Rüttler, Rosalie Ormos, Jil Cannas, Tim Hammerschick, Sarah Schlag, Walter Vetter

**Affiliations:** Department of Food Chemistry (170B), Institute of Food Chemistry, University of Hohenheim, Garbenstraße 28, D-70599 Stuttgart, Germany

**Keywords:** Countercurrent chromatography, Reversed co-current mode, Sample preparation, Vegetable oil, Sterol, Tocopherol

## Abstract

Countercurrent chromatography (CCC) is a preparative instrumental method where both the mobile and stationary phases are liquids and which are predominantly used for the isolation of natural products. In this study, we widened the scope of CCC by using it as an instrumental method for the direct enrichment of the free sterol fraction from plant oils to which they contribute with ~ 1%. For the enrichment of sterols in a narrow band, we employed the so-called co-current CCC (ccCCC) mode in which both liquid phases of the solvent system (here: *n*-hexane/ethanol/methanol/water (34:11:12:2, v/v/v/v)) are moved at different flow rates in the same direction. Different from previous applications of ccCCC, the lower and predominant “stationary” phase (LP_s_) was pumped twice as fast as the mobile upper phase (UP_m_). This novel reversed ccCCC mode improved the performance but also required a higher demand of LP_s_ compared to UP_m_. Therefore, the exact phase composition of UP_m_ and LP_s_ was determined by gas chromatography and Karl Fischer titration. This step enabled the direct preparation of LP_s_ which considerably reduced the waste of solvents. Internal standards (phenyl-substituted fatty acid alkyl esters) were synthesised and utilised to frame the free sterol fraction. This approach allowed a fractionation of free sterols based on the UV signal and compensated run-to-run variations. The reversed ccCCC method was then applied to the sample preparation of five vegetable oils. In addition to free sterols, free tocochromanols (tocopherols, vitamin E) were also eluted in the same fraction as free sterols.

**Graphical Abstract:**

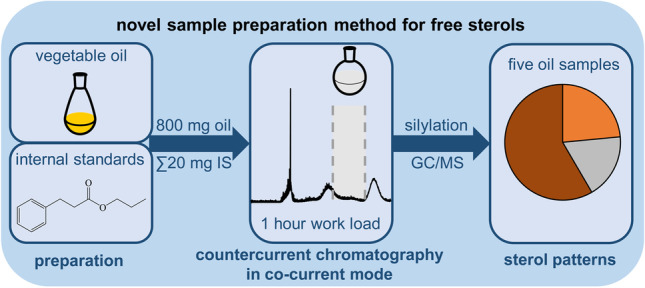

**Supplementary Information:**

The online version contains supplementary material available at 10.1007/s00216-023-04766-9.

## Introduction

Sterols are a group of structurally variable bioactive triterpenoids, which are of great importance in human nutrition [1]. Sterols are characterised by a C3-hydroxylated sterane backbone and an alkyl side chain of eight or more carbon atoms on C17 [2]. Alkyl groups, a methylene bridge and double bonds may occur in different positions of the sterane backbone or the side chain [2]. Typically, sterols contribute ~ 1% to the lipids of vegetable oils, which consist predominantly of triacylglycerols (TAGs, > 95%) [3]. Commonly, sterols are stored in free form in the lipid compartments of plants [4]. However, the hydroxyl group on C3 of the sterane backbone can also be esterified [5]. Yet, information on the real free sterol fraction is scarcely found in the scientific literature [6–9]. This is mainly due to the fact that routine methods for sterol determinations usually take advantage of the saponification of the lipid extract of samples [10]. This step first releases fatty acids from triacylglycerols followed by their precipitation in the form of soaps [11]. Hence, the bulk of the sample matrix is removed and this procedure is very effective for the enrichment of sterols. However, it also cleaves fatty acids bound to sterols (steryl esters). Thus, the free sterol fraction can only be determined by omitting the saponification step. This can be achieved e.g. by direct solid phase extraction (SPE) of vegetable oils [3, 9, 12]. Such methods may lead to the degradation of sensitive sample components [13]. Also, SPE fractionations are laborious, difficult to automate and thus little appreciated in routine analyses [6, 9].

In this study, we aimed to develop a gentle sample preparation method for the direct enrichment of free sterols from vegetable oils by means of countercurrent chromatography (CCC). CCC is an instrumental liquid–liquid chromatographic method widely used for the preparative isolation of natural products [14]. Hence, CCC was pre-destined to handle the required high sample loads given the presence of high amounts of TAGs. CCC takes advantage of a biphasic solvent system with the predominant phase kept stationary (anticipated share ~ 90%) and the other one being used as the mobile phase. Analyses take place in one or several multi-wound coils and the separation is directed by the partitioning of analytes between the stationary and mobile phase in the planetary moving CCC system. This is expressed by the partition coefficient (*K* value) [15].

CCC has already been successfully applied for the isolation of sterols from natural sources [16–18]. The major challenge of these methods is to separate structurally similar sterols [16, 19]. However, in the present case, this was not the goal. Instead, all sterols should be eluted in a rather narrow fraction but liberated from other lipid classes such as TAGs, steryl esters and free fatty acids. This can be achieved by the application of CCC in co-current mode (ccCCC) where not only the mobile phase but also the stationary phase is transported in the same direction albeit at a different flow rate [20]. Initially introduced by Sutherland et al. [21] and thoroughly described by Berthod and Hassoun [22], this special CCC mode is characterised by an accelerated elution of all analytes with comparatively small bandwidths [22]. Initial experiments indicated that the best performance could be reached by the application of ccCCC in a novel mode where the more abundant (“stationary”) phase is moved faster than the mobile phase. The associated disadvantage was that the novel reversed ccCCC mode was accompanied by a higher demand for the "stationary" phase. This was overcome by the determination of the composition of the “stationary” phase followed by its separate mixing and use. Last but not least, we aimed to introduce internal standards for the monitoring of the start and end points of the free sterol fraction.

## Materials and methods

### Solvents and chemicals

*n*-Propanol (for synthesis), squalene (99%) and a technical β-sitosterol product (55.5% β-sitosterol, 34.2% campesterol [16]) were purchased from Merck (Darmstadt, Germany). Silica gel 60 (for column chromatography), 3-phenyl propionic acid (99%), 5α-cholestane (97%), cholesterol (> 99%) and stearic acid (99%) were ordered from Sigma-Aldrich (Steinheim, Germany). Dichloromethane (99.8%), methanol (HPLC gradient grade) and *n*-hexane (HPLC gradient grade) were purchased from Th. Geyer (Renningen, Germany). Ethanol (distilled before use, 7.4% water content), sodium chloride (99%), pyridine (99%) and sulphuric acid (96%) were obtained from Carl Roth (Karlsruhe, Germany). Tripalmitin (95%, containing traces of dipalmitoylglycerol), cholesteryl stearate (97%) and elaidic acid (99%) were from Fluka (Taufkirchen, Germany). Palmitic acid (98%) was purchased from Riedel de Häen (Seelze, Germany). 8-Phenyl octanoic acid (97%) and *n*-hexanol (99%) were from Thermo Fisher Scientific (Kandel, Germany). The trimethylsilylating reagent SILYL-991 was ordered from Macherey–Nagel (Düren, Germany). Nitrogen and helium 5.0 quality originated from Westfalen company (Münster, Germany). Dihydrolanosterol [19] and α-tocopherol [23] were isolated autonomously with CCC. Demineralised water was obtained through the in-house supply (Hohenheim, Germany).

### Samples

Five vegetable oils (chili seed oil, high oleic sunflower oil, palm oil, soybean oil and corn oil) were purchased and stored at 4 °C under the exclusion of light until use. Chili seed oil was used for method development and high oleic sunflower oil, palm oil, soybean oil and corn oil for the application of the developed ccCCC method.

### Specification of the countercurrent chromatographic separation mode

According to Conway and Ito [24], *K* values were determined via shake flask experiments in a slightly modified approach [19]. In brief, the biphasic solvent system *n*-hexane/ethanol/methanol/water (34:12:12:1, v/v/v/v) was prepared in a 20 mL derivatisation tube and upper phase (UP) and lower phase (LP) were separated after equilibration (~ 10 min) at room temperature. Subsequently, a lipid stock solution (squalene, palmitic acid, elaidic acid, stearic acid, tripalmitin, dipalmitoylglycerol, cholesteryl stearate, dihydrolanosterol, cholesterol, β-sitosterol, campesterol and α-tocopherol) [13] was used for the determination of *K* values and analysed by gas chromatography with mass spectrometry (GC/MS) in selected ion monitoring mode (GC/MS-SIM) [19].

### Physical and chemical properties of the solvent system

The phase ratio of the solvent system *n*-hexane/ethanol/methanol/water (34:12:12:1, v/v/v/v) was determined by mixing 102 mL *n*-hexane, 36 mL ethanol, 36 mL methanol and 3 mL water in a 500 mL separating funnel. After phase equilibration (~ 10 min), LP and UP were drained separately into 100 mL measuring cylinders. Subsequently, densities of UP and LP were determined pycnometrically. The proportions of organic solvents *n*-hexane, ethanol and methanol in UP and LP of *n*-hexane/ethanol/methanol/water (34:12:12:1, v/v/v/v) were determined according to Englert and Vetter [25]. In brief, GC/FID measurements were performed in quintuplicate with external standard calibration to quantify the solvents in both liquid phases [25]. The water content of UP and LP was determined by Karl Fischer titration as shown elsewhere [25].

### Synthesis of phenyl-substituted fatty acid alkyl esters

Transesterification of 200 mg of 8-phenyl octanoic acid (Ph8) and 3-phenyl propionic acid (Ph3) was carried out with 4 mL of 1% sulphuric acid in *n*-hexanol (8-phenyl octanoic acid *n*-hexyl ester, Ph8-6E) or *n*-propanol (3-phenyl propionic acid *n*-propyl ester, Ph3-3E) at 100 °C for 4 h, respectively. The synthesis solutions were mixed with 2 mL *n*-hexane, 1 mL saturated NaCl solution and 3 mL demin. water. The organic phases were extracted and concentrated with a rotary evaporator (50 °C/30 mbar). The residues of the esterification were dissolved in *n*-hexane and fractionated on deactivated silica gel (5 g, 20% water, w/w) according to a modified solid phase extraction protocol of Hammann et al. [6]. Ph8-6E and Ph3-3E (~ 120 mg each) were eluted with 40 mL *n*-hexane leaving residual impurities on the solid phase.

### Countercurrent chromatography in co-current mode (ccCCC)

ccCCC was conducted with a QuikPrep MK8 instrument (AECS, London, UK). The CCC centrifuge was equipped with two dynamically mounted bobbins, both consisting of two coils as described by Rüttler et al. [19]. For practical reasons, UP and LP were additionally labelled with a subscript letter that denotes their respective use in ccCCC as mobile phase (_m_) or stationary phase (_s_), namely UP_m_ and LP_s_.

All ccCCC separations were conducted at the maximum rotor speed of 870 rpm to ensure the best possible stationary phase retention [26, 27]. Likewise, different combinations of flow rates of mobile and stationary phases were modelled in advance to ensure short separation times and suitable separation performances. Before each separation sequence, the system was purged with nitrogen and LP_s_ was pumped into coils 2 and 3 (coil volume *V*_*c*_ = 236 mL). The proportion of the stationary phase (*S*_*f*_ value) was then determined at 870 rpm and a mobile phase flow rate (*F*_*m*_) of 2 mL/min. After 10 min equilibration with UP_m_ and LP_s_ in co-current flow (*F*_*m*_ = 2 mL/min, stationary phase flow rate (*F*_*s*_) 4 mL/min), separation sequences were performed in tail-to-head mode (mobile phase flown in ascending direction) with the solvent system *n*-hexane/ethanol/methanol/water (34:12:12:1, v/v/v/v) at 870 rpm and 20 °C.

Two separation sequences were carried out with equilibrated and directly prepared liquid phases to develop the ccCCC method. Here, chili seed oil was used because it featured ~ 16% dihydrolanosterol and ~ 3% cholesterol, i.e. a very early and a late eluting sterol. In either case, ~ 800 mg of the respective vegetable oil (chili seed oil, high oleic sunflower oil, palm oil, soybean oil and corn oil) and 10 mg of the internal standards Ph8-6E and Ph3-3E were dissolved in 4.5 mL UP_m_ and LP_s_, respectively, and injected via the sample loop.

The elution of internal standards was based on the retention volume (*V*_*r*_), the volume of mobile (*V*_*m*_) and stationary phases (*V*_*s*_) in the coil, *F*_*m*_ and *F*_*s*_ and the ccCCC retention time (*t*_*r*_) (Eqs. 1–3) [20, 22].

1 $${\text{V}}_{\text{r}}\text{ = }\left({\text{F}}_{\text{m}}+ \text{ } {\text{F}}_{\text{s}}\right){\text{}}\cdot\left(\frac{{\text{V}}_{\text{m}}\text{ + }\text{K }\text{}\cdot{\text{V}}_{\text{s}}}{{\text{F}}_{\text{m}}\text{ + }\text{K }\text{}\cdot{\text{F}}_{\text{s}}}\right)$$

2 $${\text{t}}_{\text{r}}\text{ = }\frac{{\text{V}}_{\text{r}}}{{\text{F}}_{\text{m}}+ \text{ } {\text{F}}_{\text{s}}}$$

3 $${\text{K}}\text{ = }\frac{{\text{t}}_{\text{r}}{\text{}\cdot}{\text{ F}}_{\text{m }}-{\text{ V}}_{\text{m}}}{{\text{V}}_{\text{s}}\text{ - }{\text{t}}_{\text{r}}\text{}\cdot{\text{F}}_{\text{s}}}$$

For the representation of ccCCC elution volumes independent of the coil volume, a modified normalisation according to Hammann et al. [28] was performed. The corrected elution volume (CEV) was calculated using *V*_*r*_ and *V*_*m*_ and normalised to the maximum elution volume (*V*_*max*_) that is reached until the stationary phase is completely exchanged in ccCCC (Eq. 4). *V*_*max*_ was dependent on the applied flow rates *F*_*m*_ and *F*_*s*_ (Eq. 5).

4 $$\text{CEV = }\left(\frac{{\text{V}}_{\text{r }}-{\text{ V}}_{\text{m}}}{{\text{V}}_{\text{max }}-{\text{ V}}_{\text{m}}}\right)\text{}\cdot\text{100\%}$$

5 $${\text{V}}_{\text{max}}\text{ = }\frac{{\text{V}}_{\text{s }}{\text{}}\cdot\text{ (}{\text{F}}_{\text{m}}+ \text{ } {\text{F}}_{\text{s}}\text{)}}{{\text{F}}_{\text{s}}}$$

In ccCCC, UP_m_ and LP_s_ are pumped simultaneously, which is why the maximum CEV was normalised to 100%. A CEV of 100% in ccCCC corresponds to a complete exchange of the stationary phase after a certain time (*V*_*s*_/*F*_*s*_). In conventional CCC, CEV can be greater than 100%. Here, the stationary phase is not pumped through the CCC centrifuge and CEV is proportional to $\it {\text{K}} \, {\text{}\cdot}{\text{ S}}_{\text{f}}$ [29]. For example, with a theoretical *S*_*f*_ value of 100% and a *K* value of 1.5, CEV becomes 150%. When comparing the two modes, it must be taken into account that in ccCCC, all analytes must have been eluted from the coil at CEV = 100%, whereas in the conventional CCC mode, analytes elute as a function of $\it {\text{K}} \, {\text{}\cdot}{\text{ S}}_{\text{f}}$ at arbitrarily large CEVs.

Initially, TAGs and steryl esters (CEV≈20–25%) were collected in a preliminary run (fraction weight see Tab. S1). Enrichment of free sterols occurred upon the UV signal of the internal standards Ph8-6E (start signal) and Ph3-3E (stop signal) at 208 nm, which was recorded with a Flash 10 diode array detector (ECOM, Praha, Czech Republic). The injection of the next vegetable oil sample took place after the complete exchange of the stationary phase (~ 52 min). ccCCC fractions were rotary evaporated (40 °C/50 mbar), transferred with *n*-hexane/dichloromethane (8:2, v/v) to 4 mL screw cap vials and weighed after the removal of the solvent (Tab. S1). Free sterol fractions were diluted to ~ 80 µg/mL, trimethylsilylated and applied for GC/MS analysis [30].

### Gas chromatography with mass spectrometry (GC/MS)

GC/MS measurements in selected ion monitoring mode (GC/MS-SIM) for the determination of partition coefficients were carried out with a 6890/5973 GC/MSD system (Hewlett-Packard/Agilent, Waldbronn, Germany). On-column injections with 1 µL of the sample solutions were performed with a 7683 autosampler (Agilent) [31]. A ZB-1 analytical column (15 m, 0.25 mm i.d., 0.10 µm film thickness, Phenomenex, Aschaffenburg, Germany) was used at a constant helium carrier gas flow rate of 1.3 mL/min. The temperature program started for 1 min at 55 °C and was raised stepwise (i) at 10 °C/min for 10 min to 155 °C, (ii) at 5 °C/min for 17 min to 240 °C and (iii) at 7.5 °C/min for 10.7 min to 320 °C (hold time: 10 min). Injector, transfer line, quadrupole and ion source temperatures were set to 55 °C, 350 °C, 150 °C, and 230 °C, respectively. The solvent delay was set to 6 min. The GC/MS-SIM method was based on quantifier and qualifier ions for each analyte in the lipid mix in four-time windows (a–d), namely: (a) 6.0–19.3 min: *m/z* 313, *m/z* 117 (palmitic acid*), *m/z* 339, *m/z* 117 (elaidic acid*), *m/z* 341, *m/z* 117 (stearic acid*); (b) 19.3–25.3 min: *m/z* 357, *m/z* 217 (5α-cholestane, internal GC/MS standard), *m/z* 69, *m/z* 81 (squalene); (c) 25.3–32.3 min: *m/z* 395, *m/z* 500 (dihydrolanosterol*), *m/z* 458, *m/z* 129 (cholesterol*), *m/z* 502, *m/z* 237 (α-tocopherol*), *m/z* 129, *m/z* 472 (campesterol*), *m/z* 129, *m/z* 486 (β-sitosterol*); (d) 32.3–48.7 min: *m/z* 371, *m/z* 625 (dipalmitoylglycerol*), *m/z* 368, *m/z* 353 (cholesteryl stearate), *m/z* 313, *m/z* 551 (tripalmitin). Compounds labelled with an asterisk (*) were silylated.

Silylated free sterol ccCCC fractions were analysed with a 6890/5973N GC/MS system (Agilent). Splitless injections of 1 µL each were performed with a CHRONECT Robotic RTC (Axel Semrau, Sprockhövel, Germany) autosampler. A Zebron guard column with deactivated tubing (2 m, 0.25 mm i.d., Phenomenex) was linked with a PressFit connector (BGB Analytik, Rheinfelden, Germany) to an Optima 5 HT analytical column (30 m, 0.25 mm i.d., 0.25 µm film thickness, Macherey Nagel). Helium was used at a constant carrier gas flow rate of 1 mL/min. The oven temperature program started isothermal (55 °C, 1 min). The temperature was stepwise ramped (i) at 20 °C/min for 10 min to 255 °C, (ii) at 1.5 °C/min for 18.7 min to 283 °C and (iii) at 15 °C/min for 1.1 min to 300 °C (hold time: 9 min) [32]. Injector, transfer line, quadrupole and ion source temperatures were set to 250 °C, 280 °C, 150 °C and 230 °C, respectively. The solvent delay was set to 7 min. GC/MS measurements were carried out in full scan mode (*m/z* 50 to *m/z* 650).

## Results and discussion

### Specification of the countercurrent chromatographic separation mode

**Fig. 1:**
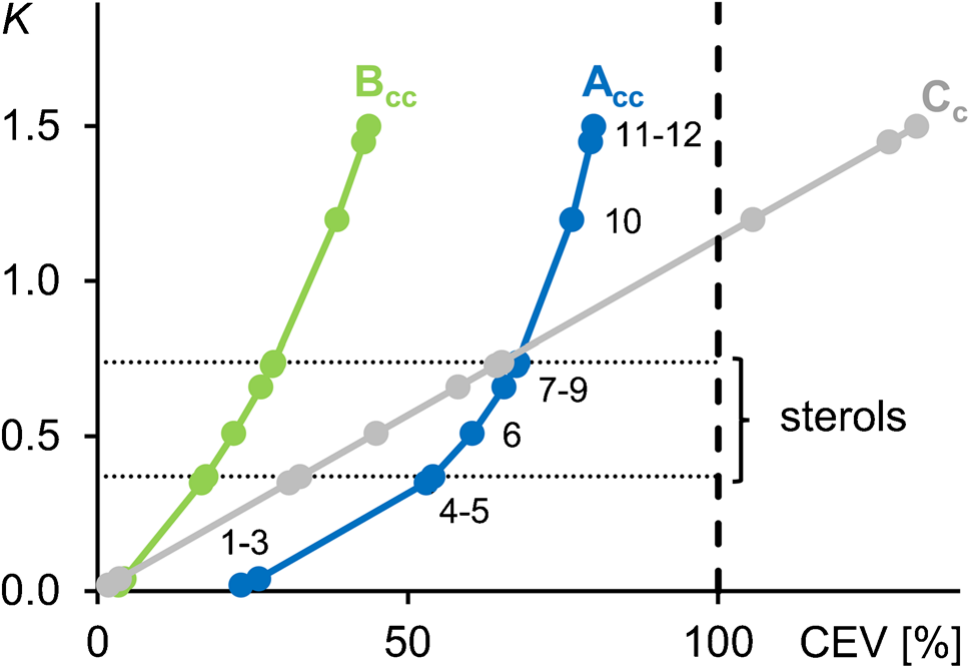
Calculated (Eq. 4) corrected elution volumes (CEVs) for fractogram A_cc_ lipids in co-current CCC at a UP flow rate *F*_*m*_ = 2 mL/min and a LP flow rate *F*_*s*_ = 4 mL/min, and fractogram B_cc_ with *F*_*m*_ = 4 mL/min and *F*_*s*_ = 2 mL/min. The fractogram C_c_ used the conventional CCC values ($\text{CEV = }\it {\text{K}} \, {\text{}}\cdot{\text{ S}}_{\text{f}}$) [29] with a coil volume of 236 mL, an *S*_*f*_ value of 88% and partition coefficients (Table 1) in tail-to-head mode. See Table 1 for compound identification

Initial tests with various systems based on* n*-hexane, ethanol/methanol and water demonstrated the usefulness of the biphasic solvent system *n*-hexane/ethanol/methanol/water (34:12:12:1, v/v/v/v) for sterols. However, according to shake flask experiments with a lipid mix (Table 1), different lipid classes showed widely varying *K* values. Similarly, to other chromatographic methods, the analytes spend most of the run time in the stationary phase. In the case of CCC with different substance (here: lipid) classes with a very wide range of *K* values, run times will become very long. This drawback can be overcome in CCC by operation in the co-current mode (ccCCC). In ccCCC, also the “stationary phase” is moved in the same direction as the mobile phase which accelerates the elution of all lipid compounds (Table 1). This is achieved by synchronous pumping of both phases with different flow rates. This was particularly evident in CEVs modelled for two ccCCC flow rate combinations compared to conventional CCC (Table 1). Specifically, the linearity in CEV according to $\it {\text{K}} \, {\text{}}\cdot{\text{ S}}_{\text{f}}$ in conventional CCC (Fig. 1, fractogram C_c_) [29] was substituted with an exponential progression with ccCCC (Fig. 1, fractogram A_cc_ and B_cc_). Therefore, elution characteristics were expressed by CEV (%) instead of *K* values (“Countercurrent chromatography in co-current mode (ccCCC)”). Tests with sterols with the highest and lowest possible equivalent chain length (ECL) [33] values in vegetable oils, i.e. dihydrolanosterol (ECL = 29) and cholesterol (ECL = 26), were used to verify that other lipid compounds in the mix were sufficiently separated from the sterol fraction. In this context, it is advantageous when the analytes are eluting in the region of the inflexion point (CEV ~ 50–70%) of the sigmoidal ccCCC elution curve since the elution volume is rapidly increasing in this range [20]. Typically, the best performance in ccCCC is obtained at flow rates of *F*_*m*_ = 4 mL/min and *F*_*s*_ = 2 mL/min [22]. However, in the equilibrated CCC system, both phases are not present at the same ratio. Traditionally, the coil is filled with the stationary phase and then the mobile phase is pumped until its breakthrough. In the present case, an *S*_*f*_ value of 88% was obtained. Since the stationary phase is present in the coils in a ~ 7.5-fold higher proportion (88:12, v/v), its flow rate in ccCCC can also be higher than the one of the mobile phase. For example, using a reversed flow rate combination of *F*_*m*_ = 2 mL/min and *F*_*s*_ = 4 mL/min, the time to pass through the stationary phase is still longer than the time to travel through the mobile phase. This reversed ccCCC mode is particularly useful for rapid sample preparation, as the exchange of the stationary phase and thus a complete elution of all added analytes is twice as fast. Specifically, fractogram A_cc_ (Fig. 1) resulted in a narrower ccCCC fractionation window of free sterols than fractogram B_cc_ (Fig. 1, time interval between the first and last eluting sterol, A_cc_: 6.8 min vs. B_cc_: 11.2 min). Moreover, the higher flow rate of the stationary phase (*F*_*s*_ = 4 mL/min and *F*_*m*_ = 2 mL/min) resulted in a time saving of 51.9 min per ccCCC run with respect to the converse flow rate combination. However, based on an *S*_*f*_ value of ~ 88%, a flow rate combination of *F*_*m*_ = 2 mL/min and *F*_*s*_ = 4 mL/min was also associated with a ~ 3.4-fold higher consumption of stationary phase LP_s_ compared with mobile phase UP_m_. Hence, the traditional way of preparing solvent systems—i.e. adding the solvent components and separating the two phases from the equilibrated solvent system—would have been linked with a notable waste of unusable UP_m_. Hence, we analysed the exact composition of LP_s_ in order to prepare it directly by mixing the individual components.

**Table 1:** Experimental partition coefficients *K* (here: between lower and upper phase, *K*_L/U_) from shake flask experiments and calculated corrected elution volumes (CEV) for flow rate combinations A (mobile phase 2 mL/min and stationary phase 4 mL/min) and B (mobile phase 4 mL/min and stationary phase 2 mL/min) in co-current CCC (ccCCC) and C with conventional CCC (CCC) with a mobile phase flow rate of 2 mL/min. CEV for ccCCC (Eq. 4) and CCC ($\text{CEV} = {\text{K}} \, {\cdot}{\text{S}}_{\text{f}}$) [26] were calculated with a coil volume of 236 mL and an *S*_*f*_ value of 88% in tail-to-head mode

Lipid	Shake flask *K*_L/U_	CEV A [%] ccCCC	CEV B [%] ccCCC	CEV C [%] CCC
Tripalmitin (1)	0.02	23.1	3.3	1.8
Cholesteryl stearate (2)	0.02	23.1	3.3	1.8
Squalene (3)	0.04	25.9	4.2	3.5
Dipalmitoylglycerol (4)	0.35	52.9	16.7	30.8
Dihydrolanosterol (5)	0.37	54.0	17.4	32.6
α-Tocopherol (6)	0.51	60.4	22.0	44.9
β-Sitosterol (7)	0.66	65.5	26.3	58.1
Campesterol (8)	0.73	67.5	28.2	64.2
Cholesterol (9)	0.74	67.7	28.4	65.1
Stearic acid (10)	1.20	76.5	38.5	105.6
Elaidic acid (11)	1.45	79.5	42.9	127.6
Palmitic acid (12)	1.50	80.0	43.7	132.0

### Saving solvent by the direct preparation of LP_s_

**Fig. 2:**
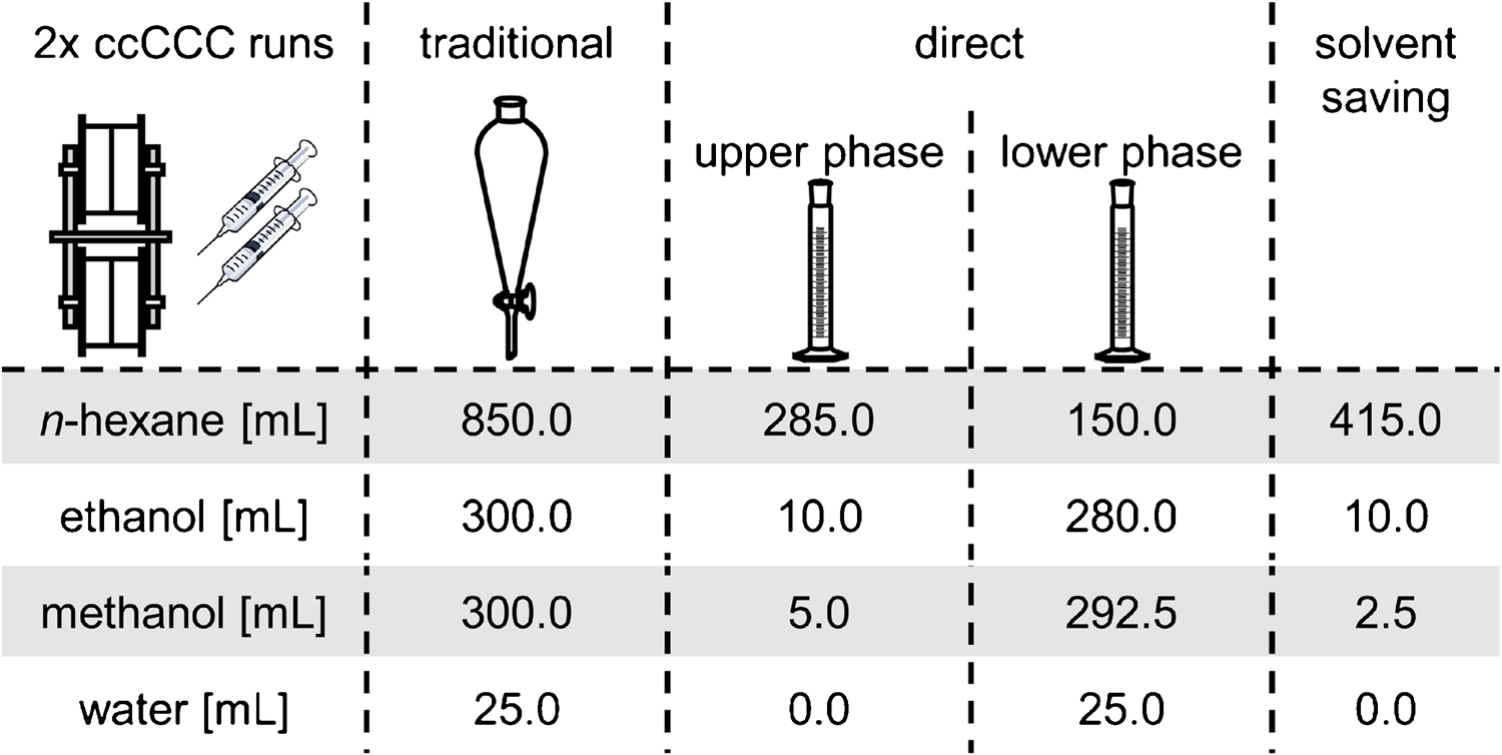
Comparison of solvent requirements for traditional preparation and direct preparation of *n*-hexane/ethanol/methanol/water (34:11:12:2, v/v/v/v) for two successive runs with CCC in co-current mode (ccCCC)

The large-scale preparation of *n*-hexane/ethanol/methanol/water (34:12:12:1, v/v/v/v) and the subsequent volumetric measurement resulted in a phase ratio of 1:1 (UP_m_:LP_s_, v/v). Both phases were separated and analysed by pycnometric density determination. The measured density of UP_m_ of 0.6655 g/mL was slightly higher than the one of *n*-hexane (0.6609 g/mL). This was in line with the expectation that UP_m_ should contain only minute amounts of methanol, ethanol and water. Conversely, the measured density of LP_s_ of 0.7822 g/mL was distinctly lower than the one of methanol (0.7919 g/mL) and ethanol (0.8064 g/mL). Thus, LP_s_ was suspected to contain higher shares of *n*-hexane. The exact phase composition was determined by GC/FID and Karl Fischer titration (“Physical and chemical properties of the solvent system”). Notably, Karl Fischer titration resulted in a water content of 6.0% in LP_s_ and 0.2% in UP_m_ (Table 2). Accordingly, the water content in LP_s_ was higher than the volume fraction used for the traditional preparation of the solvent system *n*-hexane/ethanol/methanol/water (34:12:12:1, v/v/v/v). This indicated that the hygroscopic solvents ethanol and methanol were already containing water. In agreement with that, Karl Fischer titration of ethanol (earlier distilled) and methanol (initially HPLC grade) used to prepare the solvent system resulted in water contents of 7.4% and 0.3%, respectively. Accordingly, the actual water content in the solvent system was about one part by volume higher and the ethanol content one part by volume lower than originally assumed. Consequently, the actual composition of the solvent system was corrected to *n*-hexane/ethanol/methanol/water (34:11:12:2, v/v/v/v) which was considered rather favourable, since small amounts of water are suited to stabilise solvent systems with alkanes and alcohols [32]. The use of internal standards also makes it possible to compensate for fluctuations caused by slight changes in the composition of the solvent system, especially due to storage or run-to-run variations. Surprisingly as well, the *n*-hexane content of LP_s_ amounted to 20.1% (Table 2). After these measurements, the actual solvent system *n*-hexane/ethanol/methanol/water (34:11:12:2, v/v/v/v) was prepared and the phases were separated. In parallel, LP_s_ was separately mixed according to Table 2 and combined with the separated LP_s_. These two pools were used in ccCCC. This procedure resulted in a considerable reduction of solvent consumption, especially of *n*-hexane because a much lower volume of the entire solvent system had to be prepared (Fig. 2, Table 3).

**Table 2:** Composition of the solvent system *n*-hexane/ethanol/methanol/water (34:11:12:2, v/v/v/v) in percent (± 3% standard deviation), determined by GC/FID and Karl Fischer titration

	*n*-Hexane	Ethanol	Methanol	Water
Upper phase	95.0	3.2	1.6	0.2
Lower phase	20.1	34.8	39.1	6.0

**Table 3:** Solvent consumption for one and two consecutive CCC runs in co-current mode (ccCCC). The respective amount of solvent was calculated with 52 min in co-current (cc) flow per run, with an *S*_*f*_ value of 88%, a coil volume of 236 mL and *F*_*m*_ = 2 mL/min and *F*_*s*_ = 4 mL/min in tail-to-head mode

ccCCC runs	Lower phase		Upper phase	
	Coil loading [mL]	cc flow [mL]	Equilibration [mL]	cc flow [mL]
1x	236	208	29	104
2x	236	416	29	208
∑1x	444 mL		133 mL	
∑2x	652 mL		237 mL	

### Development of the ccCCC method for the free sterol fraction

The risk of shifts in the elution volume due to slight changes in the solvent composition, *S*_*f*_ value and instrumental variations (e.g. pump) along with the virtual non-detectability of free sterols in UV detectors prompted us to introduce internal standards for marking the start and end of the free sterol fraction. Suitable internal standards were expected to be UV-active, and their *K* values should frame the free sterol fraction in ccCCC. For this purpose, several esters of phenyl alkyl fatty acids were synthesised and evaluated in ccCCC (Tab. S2). Thus, the experimental *K* values and calculated CEVs of early and late eluting dihydrolanosterol and cholesterol were used to set the fractionation frame (compounds (5) and (9) in Table 1).

**Fig. 3:**
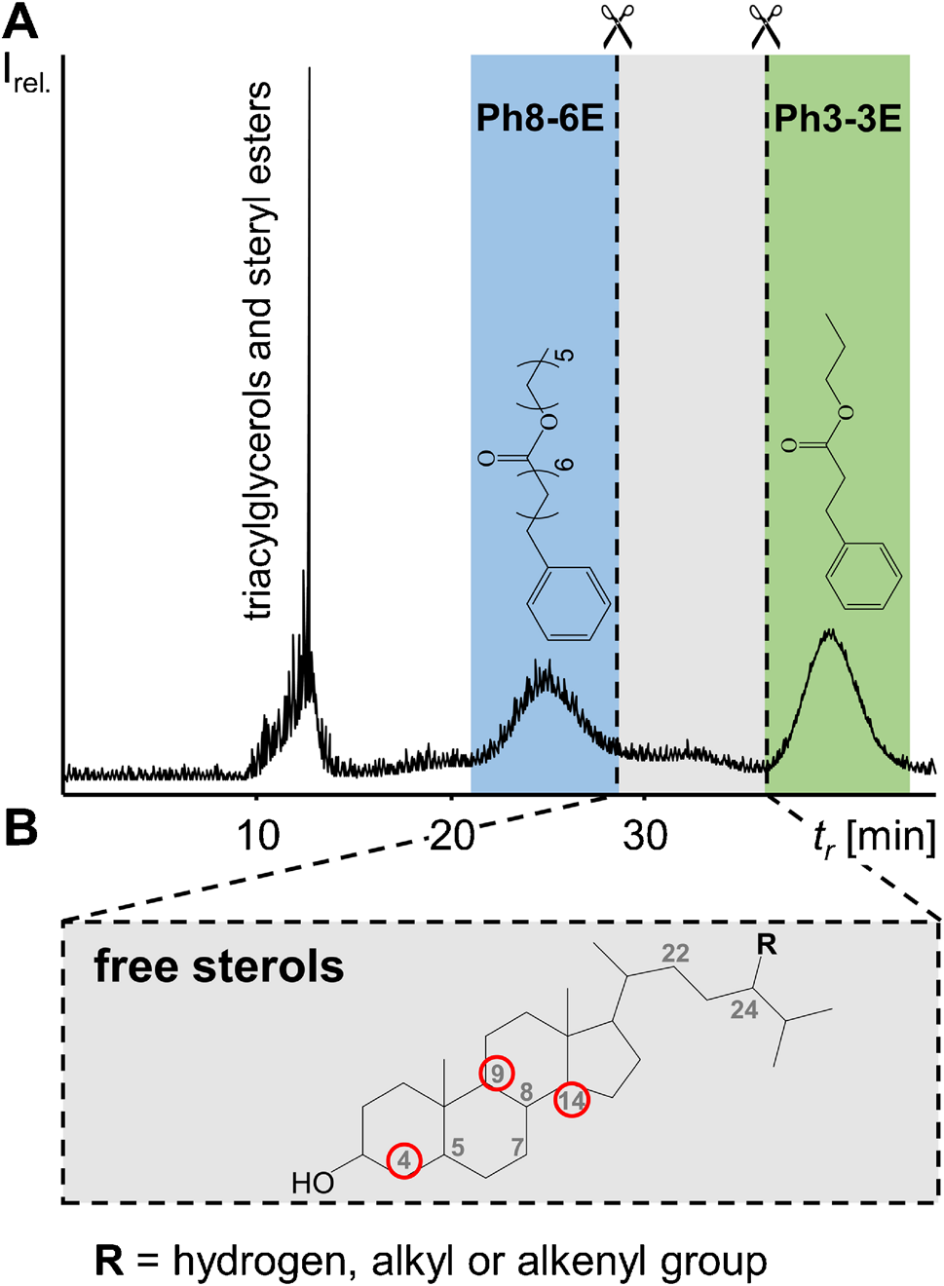
Co-current CCC UV chromatogram at 208 nm (**A**) of 800 mg chili seed oil with 10 mg each of the internal standards 8-phenyl octanoic acid *n*-hexyl ester (Ph8-6E) and 3-phenyl propionic acid *n*-propyl ester (Ph3-3E). The general chemical structure (**B**) of free sterols may include double bonds at carbon numbers 5, 7, 8, 22 and 24, as well as methyl groups at the encircled carbon numbers 4 (one to twofold methylation), and 9 or 14 (one methyl moiety each). In addition, substituent R is located in the side chain of the sterane backbone

Of all compounds tested, 8-phenyl octanoic acid *n*-hexyl ester (8Ph-6E; CEV = 42.9%) and 3-phenyl propionic acid *n*-propyl ester (Ph3-3E; CEV = 73.3%) were best suited for tagging the start and end points for the fractionation of free sterols in ccCCC (Fig. 3). Added at 10 mg each, both standards generated a prominent absorption maximum at 208 nm. Previous studies showed that the maximum tolerable sample load with the present CCC system was ~ 1 g [23]. At this amount, the solvent system gets instable which is followed by a complete loss of the stationary phase (flooding). To be on the safe side, we selected a sample load of 800 mg vegetable oil to avoid flooding (“Specification of the countercurrent chromatographic separation mode”). Repeated injections with this setup in ccCCC mode (800 mg chili seed oil, 10 mg 8Ph-6E and 3-Ph3-3E) resulted in both constant *S*_*f*_ values of 88% and CEVs of the internal standards. Accordingly, subsequent samples were injected without delay directly after 52 min (see above). This way of proceeding did not affect the CEVs of the internal standards. Also, self-prepared LP_s_ (“Saving solvent by the direct preparation of LP_s_”) was implemented without remarkable changes in the elution pattern (compared to initial tests with LP_s_ separated from the equilibrated solvent system). Accordingly, the present method (ccCCC with Ph8-6E and Ph3-3E as internal standards and self-prepared LP_s_) was found to be suited for the analysis of vegetable oils (“Fractionation of free sterols from vegetable oils with ccCCC”).

### Fractionation of free sterols from vegetable oils with ccCCC

**Fig. 4:**
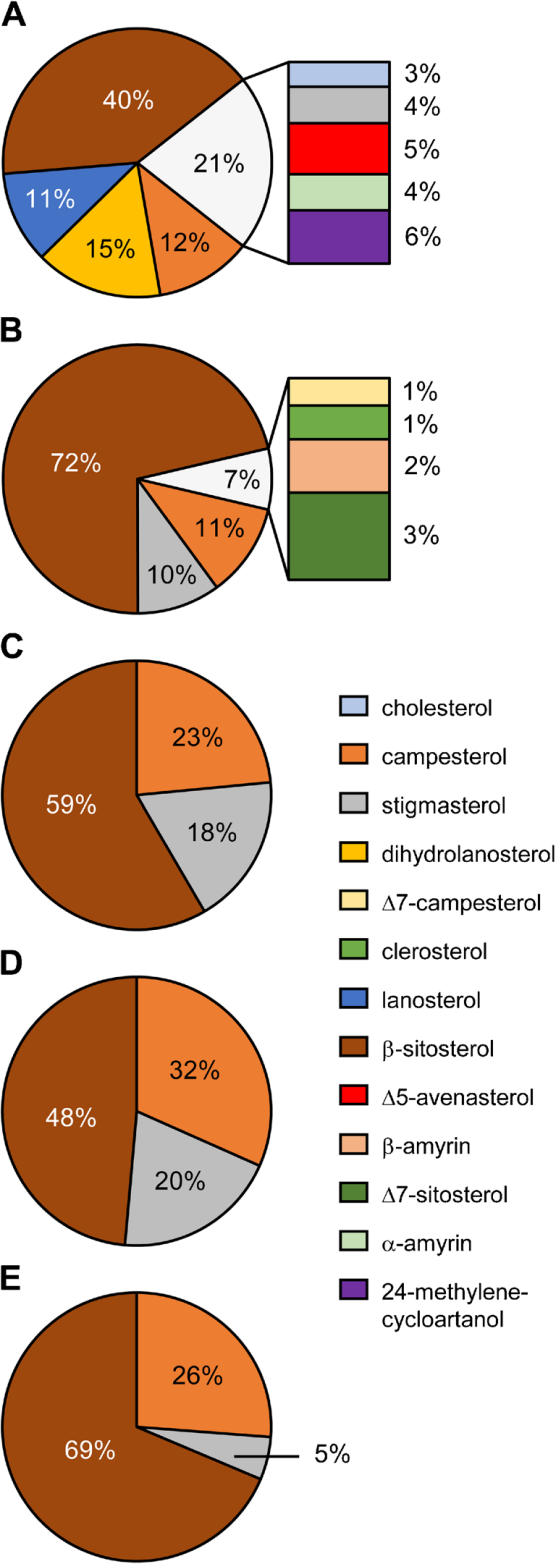
Mean distribution of free sterols (%) of **A** chili seed oil, **B** high oleic sunflower oil, **C** palm oil, **D** soybean oil and **E** corn oil after enrichment with CCC in co-current mode

The application of the ccCCC method was tested with five vegetable oils (chili seed oil, high oleic sunflower oil, palm oil, soybean oil and corn oil) analysed in duplicate. All ccCCC runs provided both constant *S*_*f*_ values of 88% and CEVs of the internal standards Ph8-6E and Ph3-3E, which underlined the good reproducibility and its suitability for routine analysis. Evaporation of the solvent, silylation and subsequent GC/MS analysis resulted in the detection of 13 sterols in total (Fig. 4). In agreement with the literature for total sterols [34], β-sitosterol was the predominant sterol in all examined oils with contributions of 40–72%. Similarly to the literature as well, palm oil, soybean oil and corn oil additionally featured prominent shares of campesterol and stigmasterol with variations within the known natural ranges which are depending on many factors (Fig. 4C–E) [34]. Additionally, four and five minor sterols < 10% were detected in chili seed oil and high oleic sunflower oil, respectively (Fig. 4A–B). These included cholesterol, stigmasterol, Δ5-avenasterol, α-amyrin, 24-methylenecycloartanol, Δ7-campesterol, clerosterol, β-amyrin and Δ7-sitosterol (Tab. S3). However, the proportion of the minor sterols was higher in chili seed oil (~ 21%) than in high oleic sunflower oil (~ 7%). Moreover, the high proportion of 15% dihydrolanosterol and 11% lanosterol in chili seed oil distinguished it from other oils measured in this study (Fig. 4A). Finally, the duplicate samples of the analysed oils provided good relative deviations of < 5% for major sterols (share > 10% of the total distribution) and < 10% for minor sterols (share < 10% of the total distribution) (Fig. S1). Thus, in addition to the system stability with multiple injections of vegetable oils with the present CCC system, a consistent enrichment and routine analysis of free sterols with ccCCC was given with virtually the same abundance ratio as with a conventional method (Fig. S2).

**Fig. 5:**
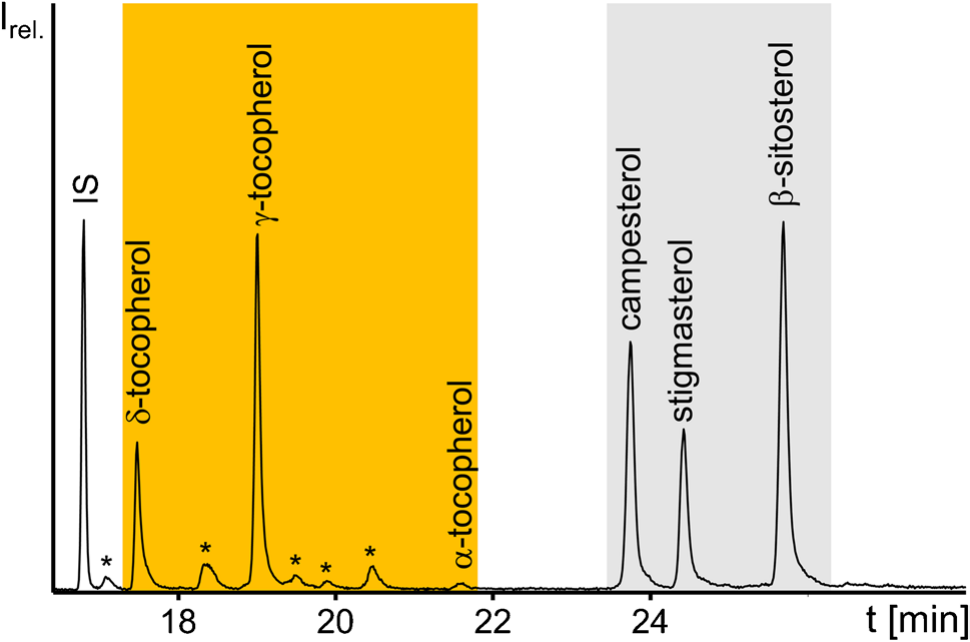
GC/MS full scan chromatogram of the trimethylsilylated free sterol fraction of soybean oil after enrichment by CCC in co-current mode. The internal standard (IS) 5α-cholestane was adjusted to 5 µg/mL. Labels: (*) steranes, (orange elution band) tocochromanols, (grey elution band) sterols

Remarkably, free tocochromanols (tocopherols, vitamin E) could also be detected at intensities comparable with free sterols by GC/MS in all sterol fractions of examined oils. This is important to note because standard sample processing protocols of tocopherols require the use of antioxidants for their protection during saponification [35, 36] which was not necessary in the present case. Silylated β- and γ-tocopherol co-eluted in the applied GC/MS method but could be distinguished by means of the ratio of *m/z* 222 to *m/z* 223 [8]. In the three relevant samples, the intensity of *m/z* 223 was more abundant than *m/z* 222, which verified that γ-tocopherol was the main compound. Specifically, free δ-tocopherol, γ-tocopherol and α-tocopherol were identified in soybean oil (Fig. 5). In addition, γ-tocopherol was qualified by GC/MS analysis in chili seed oil, α-tocopherol in palm oil and high oleic sunflower oil and γ-tocopherol and α-tocopherol in corn oil.

## Conclusions

A method was developed for the sample preparation of free sterols from vegetable oils with ccCCC in a novel reversed co-current mode. Thanks to ccCCC, it was possible to process large sample quantities (~ 800 mg vegetable oil per injection) with the solvent system *n*-hexane/ethanol/methanol/water (34:11:12:2, v/v/v/v) in separation sequences within a short period of time. Based on a typical sterol contribution of 1% to a vegetable oil, one injection will provide ~ 8 mg free sterols which is much more than usually achieved in sterol analysis [6, 9]. The faster pumping of LP_s_ compared to UP_m_ (*F*_*s*_ = 4 mL/min and *F*_*m*_ = 2 mL/min) accelerated the elution of the free sterol fraction and focused free sterols on a narrower elution band. Overall, the sample preparation time of the free sterol fraction per oil sample prior to silylation and GC/MS determination was one hour. The over-proportionally high demand for LP_s_ in the applied “reversed ccCCC mode” prompted us to directly prepare LP_s_ from its components which enabled us to save a considerable amount of solvents. The UV-active internal standards Ph8-6E and Ph3-3E, which framed the free sterol fraction in ccCCC could be monitored online. Overall, the novel ccCCC method proved to be suited for the routine analysis of free sterols in vegetable oils. Similarly, free tocochromanols could also be directly gained without the addition of antioxidants for their protection. Enrichment of free sterols with ccCCC requires fewer steps compared to SPE and is less time-consuming than SPE and conventional CCC. However, the solvent requirement for the ccCCC enrichment of free sterols is higher compared to conventional CCC and SPE. This disadvantage has been minimised through solvent reduction measures, especially for the toxic solvent hexane. Unfortunately, the use of hexane in sterol analysis as an eluent and extraction agent is well established [12] and could not be completely avoided in this study. However, the redistillation of solvents, e.g. ethanol, and also of the collected solvent waste has the potential for improving the sustainability of ccCCC.

## Supplementary Information

Below is the link to the electronic supplementary material.

Supplementary file1 (PDF 176 KB)
