# Supplementary material for: Sample preparation of free sterols from vegetable oils by countercurrent chromatography in co-current mode
Source: Anal Bioanal Chem. 2023 Jun 7;415(19):4731–40. doi: 10.1007/s00216-023-04766-9 (PMC10352415; doi:10.1007/s00216-023-04766-9)
Supplement: Supplementary file 1 — Supplementary file1 (PDF 176 KB) [file 216_2023_4766_MOESM1_ESM.pdf]

Sample preparation of free sterols from vegetable oils by countercurrent chromatography in co-current mode

Felix Rüttler[1], Rosalie Ormos[1], Jil Cannas[1], Tim Hammerschick[1], Sarah Schlag[1] and Walter Vetter[1]*

[1] University of Hohenheim, Institute of Food Chemistry, Department of Food Chemistry (170b), Garbenstraße 28, D-70599 Stuttgart, Germany

* Corresponding author

Walter Vetter

Phone: +49 711 459 24016

Fax: + 49 711 459 24377

Email: walter.vetter@uni-hohenheim.de

ORCID: 0000-0002-5592-4265

Table S1 Masses of preliminary runs (triacylglycerols and steryl esters) and free sterol fractions of vegetable oils collected with CCC in co-current mode**.**

sample	run	preliminary runs [mg]	free sterol fractions [mg]
chili seed oil	A	854	7.7
	B	783	7.2
high oleic sunflower oil	A	749	2.5
	B	766	2.6
palm oil	A	675	5.0
	B	717	6.8
soybean oil	A	719	3.7
	B	723	3.4
corn oil	A	739	4.5
	B	770	8.1

Table S2 Partition coefficients (K_{CC} values) of phenyl alkyl esters of 8-phenyl octanoic acid (Ph8) and 3-phenyl propionic acid (Ph3) with methanol (1E), *n*-propanol (3E), *n*-pentanol (5E) or *n*-hexanol (6E) with CCC in co-current mode and calculated corrected elution volumes (CEV) (**equation 4**) with flow rates of 2 mL/min (mobile phase) and 4 mL/min (stationary phase), a coil volume of 236 mL and an S_f value of 88% in tail-to-head mode.

phenyl alkyl ester	K_{CC} value	CEV [%]
Ph8-6E	0.20	42.9
Ph8-5E	0.24	45.9
Ph8-3E	0.38	54.5
Ph3-5E	0.57	62.6
Ph8-1E	0.64	64.9
Ph3-3E	1.00	73.3

Table S3 Free sterols detected by GC/MS analysis in full scan mode in (A) chili seed oil, (B) high oleic sunflower oil, (C) palm oil, (D) soybean oil and (E) corn oil after enrichment with CCC in co-current mode.

sterol	A	B	C	D	E
β-sitosterol	X	X	X	X	X
campesterol	X	X	X	X	X
stigmasterol	X	X	X	X	X
cholesterol	X				
dihydrolanosterol	X				
Δ7-campesterol		X			
clerosterol		X			
lanosterol	X				
Δ5-avenasterol	X				
β-amyrin		X			
Δ7-sitosterol		X			
α-amyrin	X				
24-methylenecycloartanol	X				

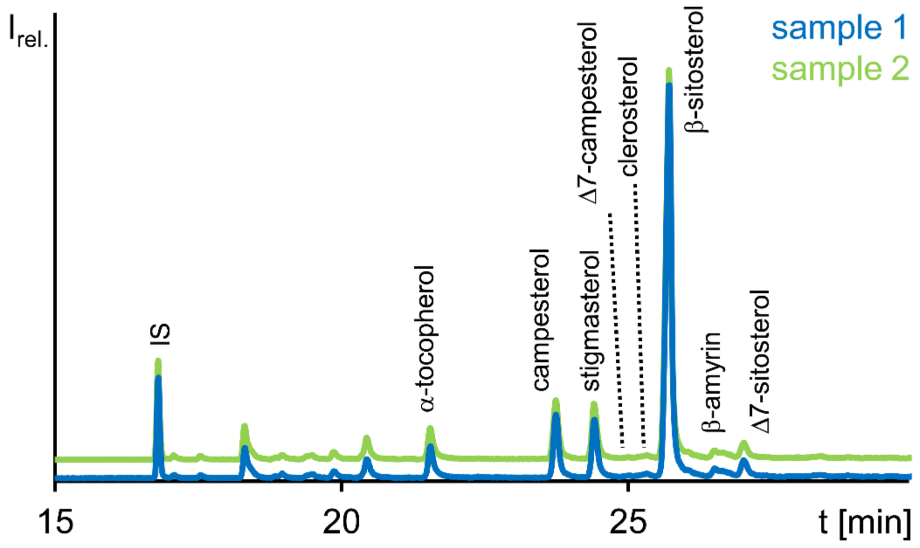

Figure S1 GC/MS full scan chromatograms of the free sterol fractions of high oleic sunflower oil samples prepared in duplicate by CCC in co-current mode.

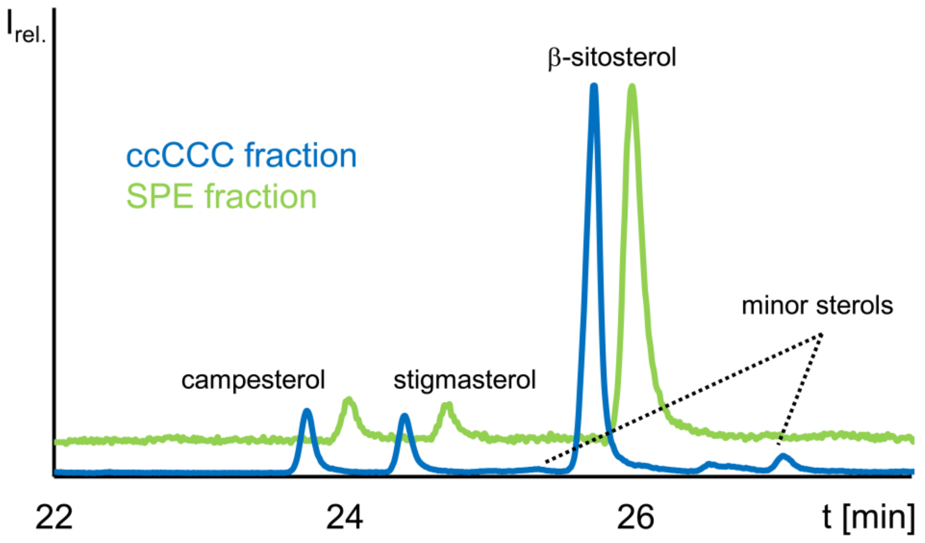

Figure S2 GC/MS full scan chromatograms (excerpts) of the trimethylsilylated sterols obtained after ccCCC (blue) and conventional cleanup including SPE (green).

SPE method parameters: high oleic sunflower oil samples of 100 mg were processed with SPE (Hammann *et al.* Lipids 2015;50:611-620). Deactivated silica gel (5 g, 20% water, *w/w*) was placed in a glass column (1 cm i.d.) and conditioned with 30 mL *n*-hexane. The sample (50 mg dissolved in *n*-hexane) was then loaded onto the column and eluted with eluents of increasing polarity. SPE fraction I (40 mL *n*-hexane): hydrocarbons; SPE fraction II (40 mL *n*-hexane/ethyl acetate, 99:1, *v/v*): esters; SPE fraction III (40 mL *n*-hexane/ethyl acetate, 95:5, *v/v*): triacylglycerols; SPE fraction IV (50 mL ethyl acetate): sterols. Specifically, SPE fraction IV was collected in 100 mL pear-shaped flasks and the solvent was removed via rotary evaporation (335 mbar/40 °C). The dry residue was transferred with *n*-hexane/DCM (8:2, *v/v*) into pre-weighed vials, weighed and taken up in 1 mL *n*-hexane. An aliquot of the sample was saponified (Schlag *et al.* Anal. Bioanal. Chem. 2022; 414:1061-1071), used for trimethylsilylation and subjected to analysis by means of GC/MS.
